# Tobacco industry’s ‘wellness’ tactic: Ethical dilemma and solutions

**DOI:** 10.18332/tpc/159119

**Published:** 2023-04-24

**Authors:** Deborah Sy

**Affiliations:** 1 Global Center for Good Governance in Tobacco Control, School of Global Studies, Thammasat University, Rangsit, Thailand

**Keywords:** ethics, tobacco industry interference, ESG, WHO FCTC Art 5.3, pharmaceutical acquisition

In recent years, tobacco companies have been investing in or acquiring pharmaceutical companies, which produce medications for a myriad of diseases, including tobacco-induced conditions and diseases, and emergency medicine. This results in ethical dilemmas – tobacco companies profit from selling medicines for health conditions, many of which are caused by tobacco products themselves. This article highlights some of these pharmaceutical acquisitions, the fundamental issues related to the same, and offers policy solutions in line with the global tobacco control treaty.

## Background

As part of the strategy to project themselves as ‘wellness companies’, major transnational tobacco companies have made some pharmaceutical investment, especially in the past 10 years.

Philip Morris International (PMI) is a majority owner of business on inhaler devices for cannabis (Syqe)^1^, inhaler products to deliver medications (Vectura)^2^, respiratory inhalers (OtiTopic)^3^, as well as oral nicotine products such as nicotine gums and pouches (Fertin Pharma, AG snus & nicotine pouches)^4,5^. In 2022, PMI also invested in COVID-19 vaccines, and owns <50% of Medicago, a Canadian biotechnology company^6^.

British American Tobacco (BAT) has a subsidiary named Reynold’s American Inc. (RAI) working on vaccines (KBio Holdings)^7^, and small shares in cannabis companies, including start-ups^8^. It has also invested in oral nicotine products such as nicotine pouches (Lyft, Velo)^9^, nicotine gums (Zonnic)^10^, and nicotine lozenges (Revel, rebranded as Velo)^11^.

Japan Tobacco International (JTI) has a pharmaceutical division^12^ that develops and sells medicines for iron and allergies, and autoimmune medicines etc. It also owns a brand of oral nicotine pouches (Nordic Spirit)^13^.

Imperial Tobacco owns two nicotine pouch brands (Skruf, zoneX)^14^. It has also invested in cannabis through two companies (Oxford Cannabinoid Technologies and Auxly Cannabis Group)^8^.

The first set of the tobacco’s acquisitions are aimed at increasing its portfolio of recreational addictive drugs and drug delivery devices; the second set involves products to treat tobacco-induced and/or related diseases; and the third set involves groundbreaking and emergency medicines, particularly vaccines – all part of a strategy to increase profits.

## Vectura: treating tobacco-induced diseases

News articles and commentators have pointed out that when the pharmaceutical company acquired by tobacco treats any of the myriad diseases caused or worsened by tobacco, then the tobacco companies’ transformation into a wellness business essentially allows them to profit from the poison and the sale of the cure. The most glaring of these examples is Vectura, which sells inhalers to patients suffering from Chronic Obstructive Pulmonary Disorder (COPD), a tobacco-induced disease^15^.

PMI acquired Vectura by buying out all outstanding stocks held by the public^16-18^, which is an expensive endeavor as the stocks were bought at a premium. It was meant to be an investment aimed at improving its corporate image, which would potentially rouse investors’ interest in PMI through increased Environment, Social and Governance (ESG) ratings^19,20^.

The repercussions were immediate – civil society, academe and others wrote to the government to challenge the acquisition of the publicly listed pharmaceutical company^21^. Two conferences, *Formulation and Delivery UK* and *Drug Delivery to the Lungs,* removed Vectura as a sponsor and participant^22,23^. Medical societies, through the European Respiratory Society^24^, and others denounced the transaction. The Forum for International Respiratory Societies issued the following statement to healthcare professionals and patients: ‘Do not use products newly developed by companies owned by the tobacco industry. Patients requesting to move away from current products of tobacco-owned companies should be given alternatives if appropriate and safe to do so, by their health care professionals’^21,25,26^. This was bolstered by a subsequently published survey which showed that 70% of patients are bothered by a tobacco company making an inhaler that treats lung conditions^17^.

## Medicago: groundbreaking vaccine

A government’s endorsement of a PMI-backed pharmaceutical, Medicago, which produces tobacco plant-based COVID-19 vaccine, also raised a pointed question: ‘Does the end of one pandemic justify the promotion of another?’^26^. The Canadian government invested in Medicago at the height of the race to end the pandemic by producing vaccines; however, during the announcement, there was no mention of Philip Morris in the news release^27^. But the very next day, Philip Morris issued its own global statement on its vaccine initiative and collaboration with the Canadian government^28,29^. And once Medicago was ready to apply to the World Health Organization’s (WHO) distribution network, Covax, to donate vaccines to foreign countries, WHO rejected the application due to its tobacco links – this pressured Medicago to discuss disinvesting with PMI^30^. WHO was decisive and quick in its response: ‘WHO and the UN have a strict policy regarding engagement with the tobacco and arms industries, so it’s likely it won’t be accepted for emergency use listing’^31^.

The Canadian government attempted to assist in resolving the issue by removing PMI as a shareholder. This was met with support from tobacco control (TC) groups who agreed that the only solution to make this vaccine useful is to remove PMI as an investor^32^.

## Due diligence and so-called corporate social responsibility

Canada’s investment in Medicago’s *Covifenz* vaccine was dubbed by a policymaker as a ‘failure of due diligence’^33^; a good reference to the due diligence required to comply with Article 5.3 of the WHO Framework Convention on Tobacco Control (FCTC), a global tobacco control treaty which urges Parties of the treaty to avoid conflicts of interest, avoid unnecessary interactions with the tobacco industry, and not give incentives to the tobacco industry for running its business^34^.

Based on corporate press releases and reporting, tobacco industry’s pharmaceutical acquisitions are part of a corporate social responsibility (CSR) strategy. In Philip Morris’ books, Medicago is promoted as a means to ‘seek impact in wellness and healthcare’, ‘using tobacco plant to become the first plant derived COVID-19 vaccine’^19,20^. Supporting or endorsing this effort boosts the tobacco industry’s capacity and credibility to sell this ‘wellness’ narrative and the related CSR stunts. It appears from the sequence of events and activities of tobacco companies globally and locally that the acquisition is part of the larger narrative of contributing to the pandemic relief. For example, in developing nations like Bangladesh and the Philippines, the tobacco industry provides tokenistic donations to compliment this narrative and, at the same time, takes the opportunity to mingle with government officials [Private communication between Global Tobacco Index Collaborators at PROGGA and HealthJustice]. Tobacco companies document these donations and incentives in their ESG reports.

ESG is a means to frame the impact of CSR activities. Investors have used ESG ratings to determine the moral and social potential of a corporation^35^. In a manner of speaking, CSR and ESG activities are two sides of the same coin. But there are global standards on ESG. United Nations Global Compact (UNGC) and United Nations Environment Program (UNEP) have an initiative to define the Principles of Responsible Investment (UNPRI), which sheds some light on how investors can better align themselves with broader objectives of society^36^. From a values perspective, there are businesses which will never align with ESG – tobacco, controversial weapons, and gambling; and according to UNPRI’s technical brief, the most commonly practiced negative screenings or exclusions in an ESG or socially conscious investment portfolio, are tobacco and weapons^37^.

The tobacco industry is aware that its business does not meet international ESG standards. Thus, it adopted a ‘transformation narrative’ to provide a counterpoint to the dogma that tobacco companies are bad. Notably, BAT’s ESG reports have a disclaimer^38^ that their report is not intended for broader audiences, but only for investors. It can be interpreted as an admission that BAT knew the material can be viewed online in many jurisdictions, by a broader audience, across the world. This is problematic because such reports represent a publicity of CSR activities in places where the same are banned or regulated. In the hands of the public, the tobacco industry’s ESG report can be deemed misleading or likely to promote the tobacco company, its brands or use of its products.

A **STOP webinar** on Tobacco Investments in the Pharmaceutical Sector brought together tobacco control leaders to look into the issue^29^. The key recommendation was to implement Article 5.3 of the tobacco control treaty and to be reminded of the fundamental and irreconcilable conflict between tobacco industry interests and public health policy. The need to incorporate Article 5.3 into law was specifically highlighted, as this would strengthen a policymaker’s position in responding to tobacco industry tactics, including isolating tobacco’s wellness investments from public health policy and governance.

To prepare for further acquisitions, governments must adopt binding policies that reflect Article 5.3 and its guidelines, including legislation that regulates both the public and private sector and covers a variety of government sectors^32^. For example, Uganda has a comprehensive section on Article 5.3 in its tobacco control law^39,29^.

In November 2021, at the ninth session of the Conference of the Parties (COP9) to the WHO FCTC, delegates expressed concern over the pharmaceutical acquisitions of tobacco companies as this hinders tobacco control implementation. The declaration asks Parties to implement the WHO FCTC, mentioning the need for increased taxation, highlighting the need to include Article 5.3 implementation in pandemic recovery plans, to support legitimate tobacco cessation services as well as to promote international cooperation to fulfill treaty obligations and counter tobacco industry interference worldwide^40^.

This **STOP Brief** stresses on the importance of applying Article 5.3 rigorously:

1. Knowing the Tobacco Industry Actors: To fulfill the mandates and objectives of the tobacco control treaty, governments must be able to identify tobacco links and relationships. This entails due diligence in looking for conflicts of interests. In legal drafting, this means, clearly defining the scope of conflict and recognizing that the term ‘tobacco industry’ should include all agents, subsidiaries, affiliates and all those receiving tobacco funding, including pharmaceutical companies that are acquired or partially funded by tobacco companies.
2. Investigating New Structures of the Tobacco Industry: Exercising due diligence also involves investigating tobacco industry mergers and acquisitions and ensuring that the tobacco industry and its subsidiaries and affiliates, which are furthering its business of promoting addictive and harmful products, do not end up being given any incentives, benefits or privileges to run their business (**Article 5.3 Guidelines**). Otherwise, tobacco companies will secure government incentives through the subsidiaries.
3. Avoiding Tobacco-linked Stocks: Placing in one company the role of both the cause of the disease and the provider of treatment, could financially incentivize the entity, causing more diseases to maximize profit – tantamount to an immoral business that is prohibited or regulated in some countries. It is important to ensure that government investments are not placed in tobacco and tobacco-affiliated stocks, as this would incentivize the tobacco industry’s overall business (**Article 5.3 Guidelines**).
4. Requiring Information: The governments should require information from the tobacco industry to ensure awareness about all the agents linked to the industry. It should also adopt a code of conduct to guide government officials on how to avoid conflicts of interest with the tobacco industry.
5. Banning so-called CSR: Governments should raise awareness about the tobacco industry’s tactics and ensure that the tobacco companies are not considered or falsely labeled ‘sustainable.’ All forms of CSR activities by the tobacco industry should be banned. (**Article 5.3, Article 13** and the corresponding **Guidelines**).
6. Protecting public officials and institutions: Governments must strengthen governance rules (such as implementing codes of conduct, limiting interactions, rejecting contributions, etc.) and exercise due diligence to protect government officials from tobacco industry interference from the tobacco industry and its affiliates and subsidiaries. They should also ensure that collaborators in the pharmaceutical industry do not include those that are funded by the tobacco industry, as that would create a conflict of interest and cast doubt on the integrity of the institutions/offices involved (**Article 5.3 Guidelines**).
7. Making the Tobacco Industry Pay: Governments must start making the tobacco industry pay for harms caused. Tobacco companies must not be allowed to get creative about new ways to deceive, harm, or kill people and profit from the same. One must take into account the harms caused by the tobacco industry to date, and the future harms it will likely cause given its history and business model^41,42^.

Article 5.3 focuses on how governments can better protect their policies from tobacco industry tactics. However, civil society also has a crucial role in treaty implementation. This includes cooperating with one another in monitoring and alerting governments (a good example is the **Global Tobacco Industry Interference Index**), setting the highest standards in conflict of interest policies, avoiding investments with tobacco, including tobacco control in the sustainable development plans, and holding the tobacco industry accountable.

Notably, STOP has started some crucial work on tobacco industry accountability and liability, including listing what TI has to be accountable for, and a **cost calculator** that sheds light on tobacco’s harms and allows tobacco control advocates to instigate discussions on tobacco taxation and liability measures.

The tobacco industry’s wellness strategy is at its infancy, it is important to nip it in the bud. The groundwork has been done through the efforts and vigilance of civil society and medical societies. The governments need to put in more work to prevent future ethical concerns. Article 5.3 of the FCTC will be the best starting point.

## Data Availability

Data sharing is not applicable to this article as no new data were created.
